# miR-22 contributes to the pathogenesis of patients with coronary artery disease by targeting MCP-1: An observational study

**DOI:** 10.1097/MD.0000000000004418

**Published:** 2016-08-19

**Authors:** Bairong Chen, Liyun Luo, Weiping Zhu, Xiaoliang Wei, Songbiao Li, Yin Huang, Mao Liu, Xiufang Lin

**Affiliations:** Department of Cardiology, The Fifth Affiliated Hospital of Sun Yat-sen University, Zhuhai, Guangdong, China.

**Keywords:** atherosclerosis, coronary artery disease, miR-22, monocyte chemoattractant protein-1, PBMC

## Abstract

The aim of this study is to determine miR-22 expression levels in peripheral blood mononuclear cells (PBMCs) of patients with coronary artery disease (CAD) and to investigate whether MCP-1 expression is regulated by miR-22. miR-22 expression in PBMCs from 60 CAD patients including stable angina pectoris (SAP) (n = 29), unstable angina pectoris (UAP) or non-ST elevation myocardial infarction (NSTEMI) (n = 17), or ST-elevation MI (STEMI) (n = 14) and 20 non-CAD subjects by real-time polymerase chain reaction (qRT-PCR). The luciferase activity assays were employed to determine whether miR-22 binds to 3′UTR of MCP-1. miR-22 mimics and inhibitors were transfected into healthy PBMCs. MCP-1 mRNA and protein levels were determined by qRT-PCR and enzyme-linked immuno sorbent assay, respectively. The qRT-PCR results showed that miR-22 levels in PBMCs were decreased in CAD patients, and MCP-1 was augmented in CAD patients and was inversely correlated with miR-22 levels. The luciferase activity assays indicated that MCP-1 was a target of miR-22. Overexpression of miR-22 could significantly repress MCP-1 expression at both mRNA and protein levels in PBMCs, whereas inhibition of miR-22 showed the opposite effects. This study revealed that miR-22 is downregulated in PBMCs from patients with CAD and that miR-22 may participate in inflammatory response by targeting MCP-1, therefore contributing CAD.

## 1 Introduction

Coronary artery disease (CAD) is one leading cause of morbidity and mortality in the world. Despite much progress in the diagnosis and treatment of this disease during the past 2 decades, CAD is still a significant public health problem.^[1,2]^ Therefore, a better understanding underlying the etiology of this disease is required. To date, atherosclerosis has been recognized to be the main pathophysiological cause of CAD. Atherosclerosis is a chronic inflammatory disease that is characterized by lipid accumulation in infiltrated immune cells within the arterial wall, creating an atherosclerotic lesion that may eventually become prone to plaque rupture.^[3]^ The pathogenesis of atherosclerosis is a result of interactions between multiple cells and a diverse set of proinflammatory factors.^[3]^ As is well known, proinflammatory factors play important roles in the formation of atherosclerotic lesions. These proinflammatory factors can induce proliferation and migration of smooth muscle cells as well as recruitment of additional immune cells, subsequently resulting in further growth of the early atherosclerotic plaque.^[4,5]^ MCP-1 is a potent chemoattractant for mononuclear cells, which is a member of the best studied CC-chemokine family. Studies have shown that MCP-1 plays a crucial role in the pathogenesis of atherosclerosis.^[6,7]^ In the pathological process of atherosclerosis, MCP-1 can promote monocytes/macrophages into early atherosclerotic lesions. The deficiency of MCP-1 can result in a significant reduction of atherosclerotic lesions in LDLR−/− mice.^[8]^ Meanwhile, in patients at risk for developing atherosclerosis, serum MCP-1 level correlates with inflammatory activity.^[9]^ Dewald et al^[10]^ observed that inhibition of MCP-1 with a neutralizing antibody leads to a decreased and delayed macrophage infiltration in the healing infarct as well as a delayed replacement of dead cardiomyocytes with granulation tissue, therefore, attenuating left ventricular function. While MCP-1 plays a key role in the initiation and development of atherosclerosis and its clinical residues, the regulatory mechanisms of MCP-1 expression are required for further investigation.

MicroRNAs (miRNAs) are a class of approximately 22 nucleotide RNAs that regulate gene expression at the posttranscriptional level. miRNAs regulate gene expression by inducing direct mRNA degradation or translational inhibition. miRNAs are involved in many physiological and pathological processes, and are associated with many diseases including atherosclerosis. Accumulating evidence indicates that miRNAs contribute to the pathogenesis of atherosclerosis mainly by regulating some pathophysiological processes, such as Vascular smooth muscle cell (VSMC) proliferation, cellular adhesion, lipid metabolism, lipid uptake and efflux, inflammatory responses, and other processes, which are strongly associated with the initiation and development of atherosclerosis.^[11–13]^ For example, inhibition of miR-221/222 can result in decreased VSMC proliferation *in vitro* and in rat arteries; miR-31 and miR-17–3p directly inhibit TNF-a induced E-selectin and ICAM-1 expression, thus controlling endothelial activation in a negative feedback loop.^[14]^ miR-30c can reduce hyperlipidemia and attenuate atherosclerosis in ApoE-/- mice by targeting microsomal triglyceride transfer protein (MTTP); miR-93 contributes to coronary atherosclerosis through targeting ABCA1 and regulating cholesterol efflux capacity^[15]^; miR-181 is a suppressor of endothelial inflammatory responses in atherosclerosis.^[16]^ Furthermore, circulating miRNAs are used for diagnostics, risk stratification, and prognosis for CAD. For example, plasma miR-145 is associated with the severity of CAD.^[17]^ Accumulating experimental evidence and clinical trials also show that miRNA are potential therapeutic targets, and the detection of circulating miRNAs might also be helpful to monitor the efficiency of a specific therapy for cardiac disease.^[18]^

MCP-1 plays a vital role in the pathogenesis of atherosclerosis and CAD. Considering important functions of MCP-1 in atherosclerosis, we attempted to investigate the regulation of MCP-1 expression by miRNAs. By using Targetscan 7.0, we predicted that MCP-1 might be one target of miR-22. To further elucidate the possible mechanism, we in the present study examined miR-22 and MCP-1 expression in peripheral blood mononuclear cells (PBMC) from patients with CAD and healthy controls and also analyzed their relationship within the same cell samples. We also examined the effect of miR-22 on MCP-1 expression and determined whether miR-22 can bind with the 3′ UTR of MCP-1.

## 2 Materials and methods

### 2.1 Patient population

A total of 80 consecutive patients undergoing diagnostic coronary angiography for chest pain evaluation at Department of Cardiology, the Fifth Affiliated Hospital of Sun Yat-sen University between January 2015 and July 2015 were recruited into this study (Table 1). Among these patients, 60 patients has been proven as CAD patients, and underwent successful percutaneous coronary intervention if needed (CAD group). 20 patients were diagnosed as non-CAD with normal coronary angiographic findings and without any atherosclerotic vascular disease (control group). Patients with CAD diagnosis were again categorized into either stable angina pectoris (SAP) (n = 29) referred to elective coronary angiography, unstable angina pectoris (UAP) or non-ST elevation myocardial infarction (NSTEMI) referred to urgent coronary angiography (n = 17), or patients with ST-elevation MI (STEMI) (n = 14) according to the ACC/AHA guidelines. The diagnosis was made at least by 2 cardiologists. All subjects including patients and controls with a history and clinical features of acute or chronic infectious disease, lung diseases, liver disease and kidney disease, malignant tumor, autoimmune diseases, and patients who took anti-inflammatory drugs were excluded from this study. Informed consent was obtained from all subjects studied in this study. This study was approved by the human ethics committee of Sun Yat-sen University.

**Table 1:**
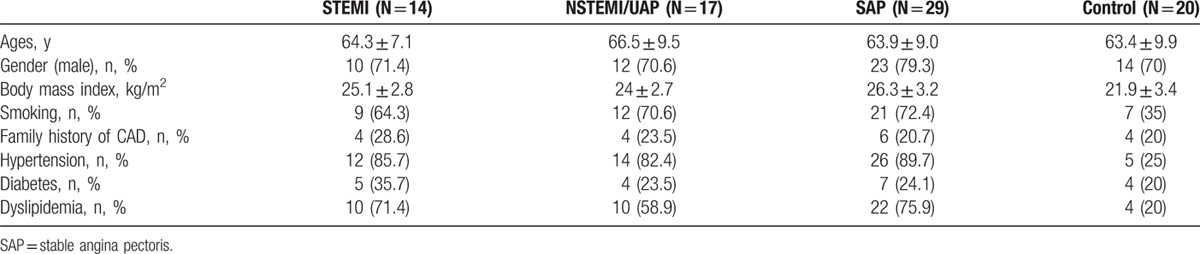
Clinical characteristics of patients with stable coronary artery disease (CAD), unstable angina pectoris/non-ST-elevation myocardial infarction (NSTEMI/UAP), or ST-elevation myocardial infarction (STEMI) referred to coronary angiography, and controls.

### 2.2 qRT-PCR for detecting miRNA and mRNA expression

PBMCs were obtained from heparinized blood by density gradient centrifugation using lymphocyte isolation liquid. Total RNA was isolated from individual PBMC from 60 CAD patients and 20 control subjects using Trizol reagents. The quantitative real-time RT-PCR analysis for the detection of miR-22 was determined by miScript SYBR Green PCR kit (Qiagen, Germany). The amplification was conducted on a Light Cycler (Biorad). The 20 μL PCR reactions consisted of 10 μL of 2 × QuantiTect SYBR Green PCR Master Mix, 2 μL of 10 × miScript Universal Primer, 2 μL of 10 × miScript Primer Assay, 1 μL of RNA, and 2 μL of RNase-free water. Mammalian U6 in the PBMCs was used to normalize the miRNA expression. The miR-22 and U6 primers were obtained from Qiagen (German). For determination of MCP-1 mRNA levels, 1 μg of total RNA for each sample were used as a template for cDNA synthesis using Reverse-Transcribe Kit (Promega Co., Madison, WI). The relative MCP-1 expression level was determined with using the following specific primers 5′-CATTGTGGCCAAGGAGATCTG-3′ and 5′-CTTCGGAGTTTGGG TTTGCTT-3′. Gene expression of the housekeeping gene GADPH was used for normalization. The PCR amplification was performed with a volume of 20 μL containing 10 μL SYBR qPCR Mix (Takara, Dalian, China). The relative expression of miR-22 and MCP-1 (defined as fold change) was calculated by the 2^−△△Ct^ (ΔCt = Ct^miR-22/MCP-1^ − Ct ^U6/GAPDH^; ΔΔCt = ΔCt^sample^ − ΔCt^conotrol^). Each sample in each group was measured in triplicate.

### 2.3 Cell culture and transfection

The isolated PBMCs were grown in RPMI 1640 medium containing 10% fetal calf serum and penicillin/streptomycin at 37°C with 5% carbon dioxide. The human embryonickidney (HEK) 293 cells in Dulbecco Modified Eagle Medium containing 10% fetal calf serum. The hsa-miR-22 mimics, hsa-miR-22 inhibitor, and unrelated sequence positive control (control mimics) and negative control (control inhibitors) were obtained from GeneCopoeia (Jiangsu, China). PBMCs were transfected by using Lipofectamine 2000 reagents in accordance with the manufacturer's instructions (Invitrogen). Total RNA was isolated from the transfected cells, and then the mRNA expression of miR-22 and MCP-1 was determined by qRT-PCR as described earlier. The protein expression of MCP-1 in supernatants was detected by enzyme-linked immuno sorbent assay (ELISA) (R&D Systems, Minneapolis, MN).

### 2.4 ELISA

MCP-1 protein levels were determined in serum or supernatants by using commercially available ELISA kits (R&D systems) according to the manufacturer's instructions.

### 2.5 Luciferase assays

Wild-type 3’ UTR of MCP-1 containing predicted target sites of miR-22 was cloned into a pMIR-REPORT miRNA expression reporter vector (Invitorgen) by using synthesized fragments. The corresponding mutant seed sequence was also constructed with the fragments. For luciferase reporter assays, HEK293 cells were transfected with miRNAs mimics or inhibitors and luciferase reporter vector. The pMIR-REPORT-β -gal control vector was also transfected as an internal control. Then, the cells were determined by using a Dual-Luciferase Reporter Assay System (Promega).

### 2.6 Statistical analysis

All statistical analyses were conducted with SPSS 16.0 software (SPSS Inc., Chicago, IL, USA). Continuous variables were expressed as mean ± SD, and were compared using Student test, or 1-way ANOVA. The determination of correlation between different variables was performed by Pearson correlation coefficient. *P* value less than 0.05 was considered to be statistically significant.

## 3 Results

### 3.1 Basic clinical characteristics of study population

This study included 60 CAD patients and 20 non-CAD individuals in total. Patients with CAD diagnosis were categorized into stable angina pectoris (SAP, n = 29), UAP/NSTEMI (n = 17), or STEMI (n = 14). All the clinical characteristics of study populations in this study are summarized in Table 1.

### 3.2 miR-22 is decreased in PBMC from patients with CAD

**Figure 1:**
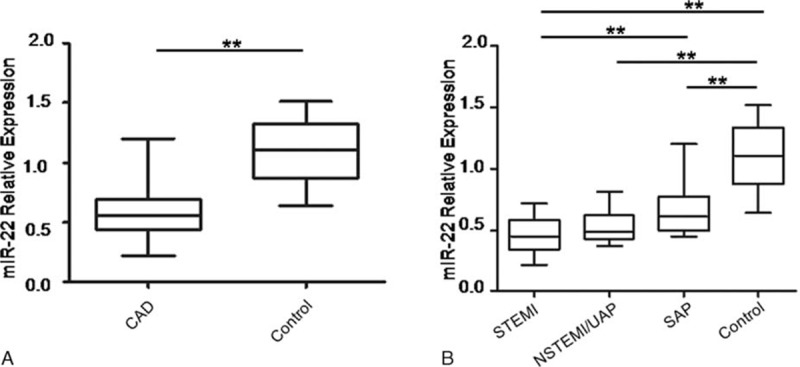
miR-22 is downregulated in PBMCs from patients with CAD. (A) miR-22 expression levels in PBMCs from CAD patients (n = 60) and controls (n = 20). (B) miR-22 expression levels in PBMCs from controls (n = 20), patients with SAP (n = 29), UAP/NSTEMI (n = 17) and STEMI (n = 14). miR-22 expression levels were quantified by qRT-PCR and data are given in relation to U6. Data are presented as mean and SD. ^∗∗^*P* < 0.001. CAD = coronary artery disease, NSTEMI = non-ST elevation myocardial infarction, PBMC = peripheral blood mononuclear cells, qRT-PCR = real-time polymerase chain reaction, SAP = stable angina pectoris, STEMI = ST elevation myocardial infarction, UAP = unstable angina pectoris.

Firstly, we measured the expression levels of miR-22 in PBMCs isolated from patients with CAD and non-CAD controls by real-time PCR. As shown in Fig. 1A, miR-22 expression levels were decreased in PBMCs from CAD patients when compared with that of healthy controls. Furthermore, we analyzed the expression levels of miR-22 in different groups of CAD, including STEMI, UAP/NSTEMI and SAP. The results showed that the expression levels of miR-22 were lower in STEMI or UAP/NSTEMI patients compared with that of SAP, but no statistical difference was observed between STEMI and UAP/NSTEMI group (Fig. 1B).

### 3.3 MCP-1 is increased in patients with CAD

**Figure 2:**
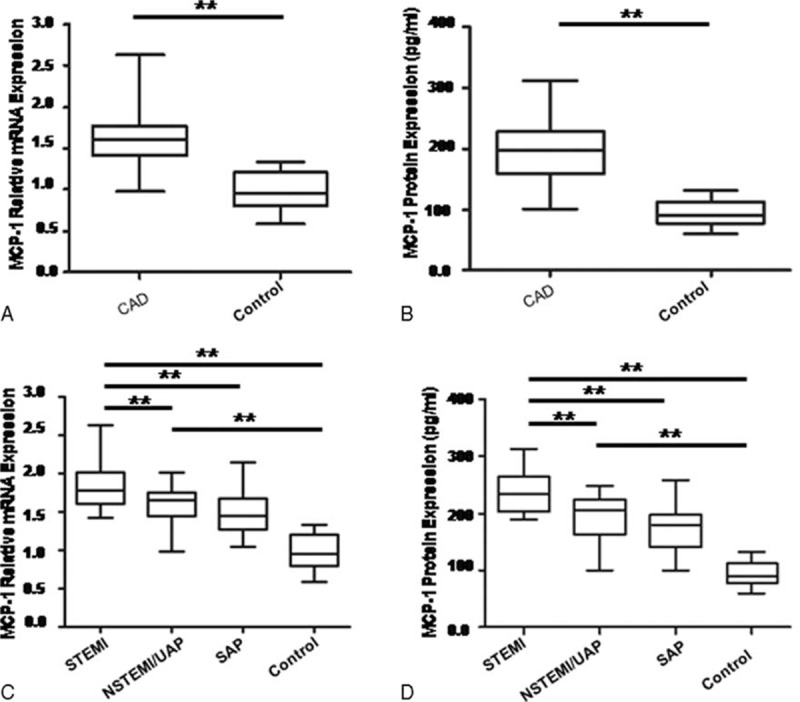
MCP-1 is increased in patients with CAD. MCP-1 miRNA (A) and protein (B) expression levels from CAD patients (n = 60) and controls (n = 20). MCP-1 mRNA (C) and protein (D) expression levels from controls (n = 20), patients with SAP (n = 29), UAP/NSTEMI (n = 17) and STEMI (n = 14). MCP-1 mRNA expression levels in PBMCs were quantified by qRT-PCR and data are given in relation to GAPDH. MCP-1 protein expression levels in serum were determined by ELISA assays. Data are presented as mean and SD. ^∗∗^*P* < 0.001. CAD = coronary artery disease, MCP-1 = monocyte chemoattractant protein-1, PBMC = peripheral blood mononuclear cells, qRT-PCR = real-time polymerase chain reaction, SAP = stable angina pectoris, UAP = unstable angina pectoris, NSTEMI = non-ST elevation myocardial infarction, STEMI = ST elevation myocardial infarction.

To evaluate whether MCP-1 expression was increased in CAD, we also determined the MCP-1 mRNA and protein levels in CAD patients and non-CAD controls. As expected, MCP-1 mRNA and protein levels were significant higher in CAD patients than those of controls (Fig. 2A and 2B). We analyzed the expression levels of miR-22 in different groups of CAD, and found that MCP-1 expression in patients with STEMI were higher than that of UAP/NSTEMI or SAP patients (Fig. 2C and 2D).

### 3.4 miR-22 is negatively correlated with MCP-1

**Figure 3:**
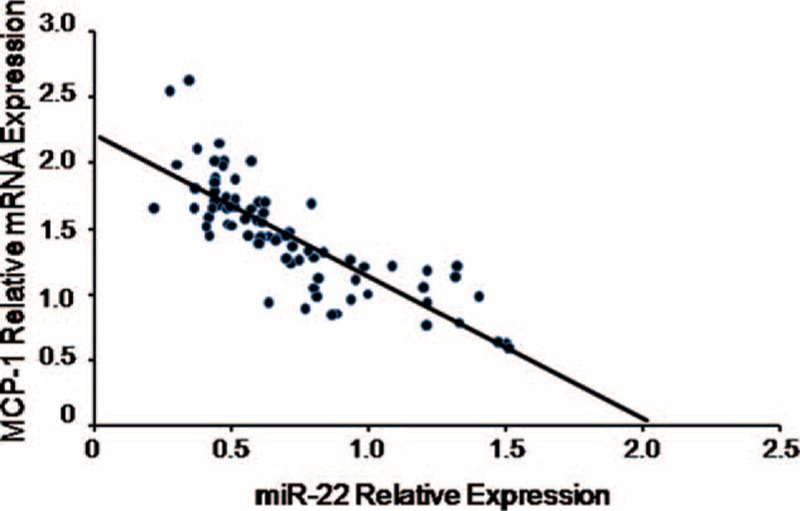
miR-22 is negatively correlated with MCP-1 mRNA levels in the whole population including CAD patients and non-CAD subjects. CAD = coronary artery disease, MCP-1 = monocyte chemoattractant protein-1.

Then, we analyzed the relationship between miR-22 expression and MCP-1 mRNA levels in PBMCs from the whole subjects in this study. As shown in Fig. 3, miR-22 were negatively related with MCP-1 mRNA expression levels (*r* = −0.816, *P* = 0.000).

### 3.5 MCP-1 is one direct target of miR-22

**Figure 4:**
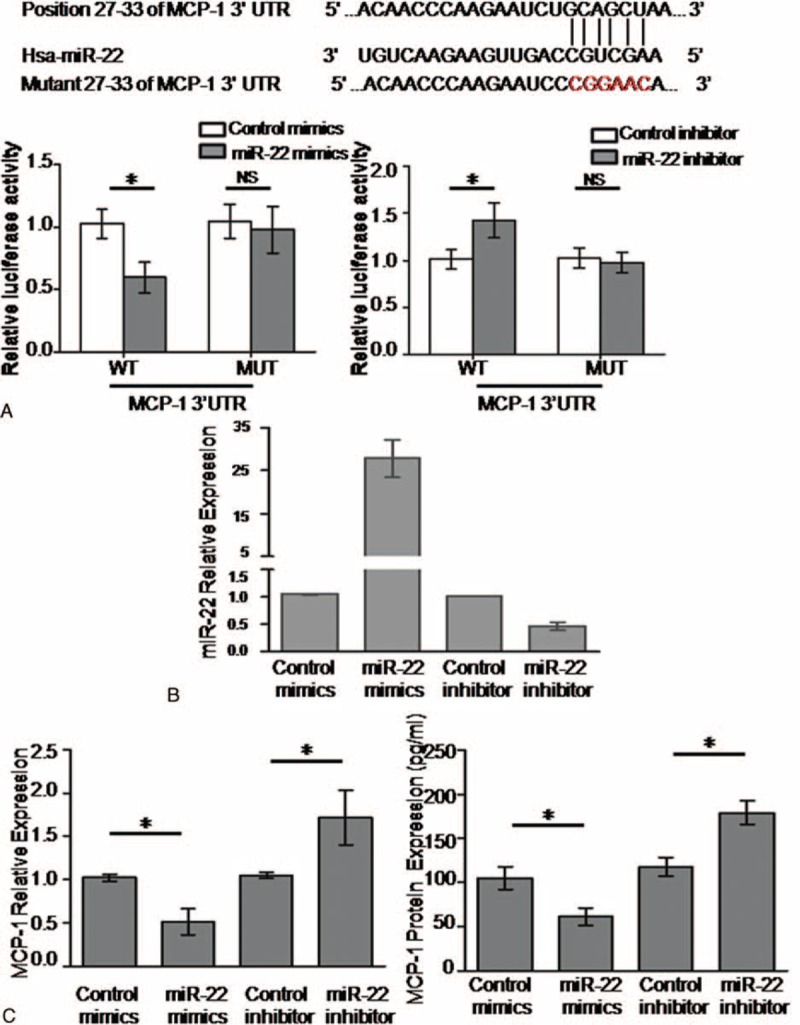
MCP-1 is a direct target of miR-22. (A) Up: potential sites in MCP-1 3′UTR binding to miR-22. The 3′UTR fragment of MCP-1 including the potential sites were cloned into a luciferase reporter vector. Mutated sequences were also generated in the seed regions to abolish binding of miR-22. Down: miR-22 mimics or inhibitor was transfected into HEK293 cells with luciferase reporter constructs harboring MCP-1 or mutant MCP-1 3′UTR fragments. The luciferase reporter assays were performed 48 hours after transfection. The luciferase activities were detected and normalized to a renilla luciferase activity. (B) miR-22 expression levels were detected followed by transfection of miR-22 mimics or inhibitor. (C) MCP-1 mRNA and protein levels after transfection with miR-22 mimics or inhibitor were assessed by qRT-PCR and ELISA. ^∗^*P* < 0.05. MCP-1 = monocyte chemoattractant protein-1, qRT-PCR = real-time polymerase chain reaction.

By using Targetscan 7.0, we found that MCP-1 may be one possible target of miR-22. To further confirm it, luciferase assays were performed. HEK293 cells were cotransfected with MCP-1 3′-UTR construct (WT) or its mutant (MUT) and miR-22 mimics or miR-22 inhibitors, followed by luciferase reporter assays (Fig. 4A). The results showed that miR-22 mimics decreased the activity of MCP-1 translation, whereas miR-22 inhibitor increased MCP-1 translation (Fig. 4A). Meanwhile, the luciferase activity of the MCP-1 mutant was not affected by the transfection of miR-22 mimics or inhibitor (Fig. 4A). The results revealed that miR-22 might bind with the 3′ UTR of MCP-1. Next, we attempted to determine the effect of miR-22 on MCP-1 expression. We firstly assessed the efficiency of transfection of miR-22 mimics and inhibitor in PBMCs. As shown in Fig. 4B, transfection of miR-22 mimics into PBMCs significantly increased its expression, whereas miR-22 inhibitor downregulted its expression level. We then detected MCP-1 mRNA and protein expression in transfected cells by qRT-PCR and ELISA. The results revealed that MCP-1 expression levels were decreased in PBMCs transfected with miR-22 mimics, when compared with the cells transfected with control mimics, whereas MCP-1 expression was significantly increased in PBMCs transfected with miR-22 inhibitors compared with the cells transfected with control inhibitors (Fig. 4C).

## 4 Discussion

In the present study, we demonstrated that miR-22 expression is significantly lower in PBMCs from CAD patients compared with that of non-CAD controls. Also, in our study we observed that MCP-1 expression is markedly increased in CAD and it is negatively correlated with miR-22. Furthermore, experimental evidence proved that miR-22 regulates MCP-1 expression by binding with its 3′UTR.

Proinflammatory factors play important roles in the pathogenesis of atherosclerosis and CAD, among which, MCP-1 has been proven to have a crucial role in the initiation and development of the diseases.^[5,8–10]^ MCP-1 can recruit blood monocytes into the arterial subendothelium, and promote the information of atherosclerotic lesions. It has been shown that the deficiency of MCP-1 in LDLR−/− mice leads to a significant reduction of the atherosclerotic lesions.^[8]^ In accordance with the findings obtained in animal models, we in the present study demonstrated that MCP-1 was significantly increased in CAD patients, which revealed that an abnormal MCP-1 expression in CAD patients may contribute to the pathogenesis of atherosclerosis and CAD. Dewald et al^[10]^ found that inhibition of MCP-1 with a neutralizing antibody leads to a decreased and delayed macrophage infiltration in the healing infarct as well as a delayed replacement of dead cardiomyocytes with granulation tissue, therefore, attenuating left ventricular function. In our study, within the CAD group, we observed that patients presenting with STEMI had significantly higher MCP-1 levels compared with SAP and UA/NSTEMI patients. It implied that increased MCP-1 may predispose stable CAD patients to plaque vulnerability and rupture and thus ACS.

miRNAs play important roles in the pathogenesis of atherosclerosis and CAD. miR-181, miR-93, miR-31, miR-17–3p, and miRNA-145 have been reported to be involved in atherosclerosis and CAD.^[3,14–17]^ miR-22 is a 22-nucleotide noncoding RNA, which is located on chromosome 17p13. miR-22 was originally identified as a tumor suppressor miRNA in many cancers.^[19]^ Lin et al^[20]^ demonstrated that miR-22 is downregulated in the synovial tissue of RA patients and negatively regulates Cyr61 expression, therefore inhibiting RA FLS proliferation and IL-6 production. By using Targetscan 7.0, we identified that MCP-1 may be one possible target of miR-22. Considering the crucial role of MCP-1 in atherosclerosis and CAD, we speculated that miR-22 may be implicated in the atherosclerosis. In this study, we demonstrated that miR-22 is downregulated in the PBMCs of patients with atherosclerosis, implying that miR-22 might play an important role in the pathogenesis of atherosclerosis. Furthermore, the expression levels of miR-22 were found to be lower in STEMI or UAP/NSTEMI patients compared with that of SAP, which further suggested that miR-22 is correlated with the severity of CAD, and it might serve as biomarkers of CAD development and progression. And it also implied that downregulated miR-22 is likely to predispose stable CAD patients to plaque vulnerability and rupture and thus ACS. Futhermore, our experimental results showed that miR-22 binds to 3′UTR of MCP-1. We also observed that overexpression of miR-22 could significantly repress MCP-1 expression at both the mRNA and protein levels, whereas inhibition of miR-22 has the opposite effects. This finding, to some extent, suggested that miR-22 may regulate MCP-1 in CAD. Decreased miR-22 in PBMCs of CAD patients may promote the expression of MCP-1 and regulate inflammatory response, therefore, affecting atherosclerotic plaque formation and instability, and contributing to the development of CAD.

Our study had several deficiencies. Firstly, it was a single-center study, which involved a small sample size. Therefore, multicenter trials are also needed to gain profound insight into the pathophysiological importance of miR-22 and determine its potential to become biomarkers in CAD. Secondly, in this study miR-22 circulation has not been detected, and so some studies are also required to determine its contributions to CAD. Thirdly, although anti-inflammatory effect of miR-22 was demonstrated through MCP-1 inhibition in CAD, further studies on the direct effect of miR-22 on atherosclerosis will be of major importance to prove any causal role of miR-22 in the development and progression of atherosclerotic disorders.

In summary, this study is the first to demonstrate that miR-22 is downregulated in PBMCs of patients with CAD. In addition, this study proves that miR-22 regulates MCP-1 expression by targeting its 3′UTR. Our findings suggest that miR-22 might may regulate systemic inflammation in CAD at least partly through its effect on MCP-1. However, these results do not provide any causal relationship between miR-22 and atherogenesis. Therefore, further studies are needed to investigate the pathophysiological role of miR-22 in atherosclerosis and CAD.

## Acknowledgments

Bairong Chen and Liyun Luo are responsible for the experiments. Weiping Zhu, Xiaoliang Wei, Songbiao Li, Yin Huang, and Mao Liu are responsible for data statistic, and Xiufang Lin is responsible for the whole design and paper writing. All authors provide final approval of the version to be submitted.
